# Protective effect of EDTA preadministration on renal ischemia

**DOI:** 10.1186/1471-2369-7-5

**Published:** 2006-03-15

**Authors:** Chiara Foglieni, Alessandro Fulgenzi, Paolo Ticozzi, Fabio Pellegatta, Clara Sciorati, Daniela Belloni, Elisabetta Ferrero, Maria Elena Ferrero

**Affiliations:** 1 Cardiovascular Department, Istituto Scientifico San Raffaele, via Olgettina, 60 Milan, Italy; 2 Istituto di Patologia Generale, Università degli Studi di Milano, via Mangiagalli 31, Milan, Italy; 3 Istituto di Scienze Farmacologiche Università degli Studi di Milano, Via Balzaretti 22, Milan, Italy; 4 Laboratory of Tumor Immunology, Istituto Scientifico San Raffaele, Via Olgettina 60, Milan, Italy

## Abstract

**Background:**

Chelation therapy with sodium edetate (EDTA) improved renal function and slowed the progression of renal insufficiency in patients subjected to lead intoxication. This study was performed to identify the underlying mechanism of the ability of EDTA treatment to protect kidneys from damage.

**Methods:**

The effects of EDTA administration were studied in a rat model of acute renal failure induced by 60 minutes ischemia followed or not by 60 minutes reperfusion. Renal ischemic damage was evaluated by histological studies and by functional studies, namely serum creatinine and blood urea nitrogen levels. Treatment with EDTA was performed 30 minutes before the induction of ischemia. Polymorphonuclear cell (PMN) adhesion capability, plasmatic nitric oxide (NO) levels and endothelial NO synthase (eNOS) renal expression were studied as well as the EDTA protection from the TNFα-induced vascular leakage in the kidneys. Data was compared by two-way analysis of variance followed by a post hoc test.

**Results:**

EDTA administration resulted in the preservation of both functional and histological parameters of rat kidneys. PMN obtained from peripheral blood of EDTA-treated ischemized rats, displayed a significant reduction in the expression of the adhesion molecule Mac-1 with respect to controls. NO was significantly increased by EDTA administration and eNOS expression was higher and more diffuse in kidneys of rats treated with EDTA than in the controls. Finally, EDTA administration was able to prevent in vivo the TNFα-induced vascular leakage in the kidneys.

**Conclusion:**

This data provides evidence that EDTA treatment is able to protect rat kidneys from ischemic damage possibly through the stimulation of NO production.

## Background

Chelation therapy with sodium edetate (EDTA) has been successfully used to treat chronic lead intoxication [1,2]. More specifically, in patients affected by chronic renal insufficiency due to environmental lead exposure, EDTA chelation therapy improved renal function and slowed the progression of renal insufficiency [3]. The mechanism by which lead-chelation therapy with EDTA delayed renal damage is unknown. Chelation with another chelating agent, the dimercaptosuccinic acid (DMSA) improved renal function and was efficacious in treating nephropathy [4] and hypertension [5], both induced in animals by long-term exposure to low-levels of lead. It has been proposed that chronic, low-level lead exposure may increase the levels of reactive oxygen species (ROS), responsible for nitric oxide (NO) inactivation [6]. Indeed, lead-chelation therapy might reduce the levels of ROS, associated to NO inactivation, and thus enhance the availability of vascular NO, potentially improving renal function and reducing hypertension [4-6]. Moreover, a multifunctional antioxidant activity has been shown for an iron chelating agent, the N,N'-bis (2-hydroxybenzyl) ethylendiamine-N,N'-diacetic acid (HBED) [7]. We asked if EDTA treatment in rats was able to reduce the renal damage, when not provoked by lead exposure. Indeed, in the present work we have studied the effect of EDTA treatment in preventing rat kidney acute damage following ischemia (Isc) or ischemia/reperfusion (Isc/R) [8,9].

We assessed the effect of EDTA systemically administered in rats, before the induction of renal Isc or Isc/R. Functional and histological kidney alterations and rat plasmatic levels of NO were evaluated, given that NO availability has been found to be responsible for the increased renal function [4,6]. In addition, being NO able to control leukocyte adhesion [10], we determined the expression of the adhesion molecule Mac-1 (monocyte chemoattractant protein-1) (CD18/CD11b) on polymorphonuclear cells (PMN) isolated from control and EDTA-treated rats. In this context, it has been shown that PMN are able to play an important role as mediators of reperfusion injury [11,12]. Finally, since endothelial NO production is an indicator of well functioning endothelium [10], we have evaluated the effect of EDTA in TNFα-induced vascular leakage in rat kidneys.

Herein we show that a single administration of EDTA results in the preservation of renal function and in the prevention of tissue damage induced by ischemic injury. In addition, we demonstrate that the preventive block of NO synthesis abrogate the protective effect of EDTA against renal ischemic damage.

## Methods

The investigation conforms with the *Guide for the Care and Use of Laboratory Animals *published by the US National Institute of Health (NIH publication NO.85-23, revised 1996), according to the animal welfare regulations of the Italian local authorities.

### Animals

Male Sprague-Dawley rats weighing about 200 g were used (Charles River Italia, Lecco, Italy) and were allowed water and standard rat chow ad libitum. All the rats were maintained at 22 ± 1°C with a 12/12 hours light/dark cycle.

### Ischemia/Reperfusion (Isc/R) model

The rats were anesthetized with an inhaled anesthesia mixture of halothane 2% (Hoechst, Milano, Italy) and oxygen. They were placed on a temperature-regulated table (38°C) (Ugo Basile, Comerio, Lecco, Italy) to maintain body temperature. Kidney ischemia (Isc) was induced by clamping the right renal artery and the right renal vein for 60 minutes with a microsurgical clamp. In the Isc/R group, at the end of the I period, the vascular clamp was removed and reperfusion of 60 minutes was performed. During the surgical procedure the heart rate and the mean arterial blood pressure (MABP) were monitored.

At the end of Isc or of Isc/R, blood samples were obtained by exanguination of rats at the aorta bifurcation level and kidneys were collected and processed for different studies. Blood and kidneys from EDTA-treated-not-ischemized rats were collected 90 minutes after EDTA administration (corresponding to 30 minutes EDTA pre-treatment+60 min Isc).

### Measurement of mean arterial blood pressure

The right femoral artery was cannulated through a polyethylene catheter and connected to a pressure transducer for the measurement of MABP [15,16]. The data was collected continuously by means of a computer and were calculated at baseline, at the end of EDTA pre-treatment (e.g 30 minutes after EDTA intravenous injection), at the end of Isc and at the end of postischemic R. In sham-operated rats the values were calculated 90 minutes after EDTA pre-treatment.

### EDTA treatment

EDTA (calcium disodium EDTA) (Collalto, Brescia, Italy) used in human therapy was employed [3], and at the same dosage (e.g. 40 mg/kg body weight). The sterile drug solution of 2 g/10 ml was opportunely diluted in physiological saline and administered by left intrafemoral vein slow infusion.

### L-NAME treatment

The inhibitor of NO synthases L-NAME [N(omega)-nitro-L-arginine methyl ester], when required, was injected simultaneously with the EDTA through the intrafemoral vein at the dose of 30 mg/kg body weight, 30 minutes before the induction of Isc or Isc/R.

### Experimental groups

The rats were randomly allocated to 4 study groups, each composed of 15 rats: group 1, controls; group 2, sham operated: the rats underwent the same surgical procedure, except that the clamp was not applied; group 3, Isc: ischemia was induced for 60 min; group 4, Isc/R: ischemia was induced for 60 min, followed by 60 min reperfusion at room temperature. Other identical 4 groups were studied, in which EDTA treatment was performed. In groups 3 and 4 intrafemoral injection of physiological saline 30 minutes before clamping was performed. The 3 and 4 EDTA-treated groups received a single intravenous injection of EDTA 30 minutes before clamping. In groups 1 and 2 intrafemoral injection of physiological saline or EDTA was performed 90 minutes before kidney removal (= 30 minutes EDTA pre-treatment+60 min Isc).

To take in consideration that EDTA could lead to increase in NO plasmatic levels through increase in eNOS expression, we further performed histological evaluations on two additional groups of rats, to verify whether the eNOS inhibitor L-NAME was able to block the protective effect of EDTA in renal ischemic injury. In such groups the animals were simultaneously treated with EDTA and L-NAME 30 minutes before the induction of Isc (group 5) and 30 minutes before the induction of Isc/R (group 6).

### Functional studies

Serum creatinine was measured using a modified Jaffe's reaction, and blood urea nitrogen was measured on the AEROSET system (Abbott Laboratories, Abbott Park, IL) [17].

### Histopathology and immunofluorescence microscopy

Kidneys were excised, decapsulated, dissected into 4 pieces along the major ax, fixed by immersion in 4% paraformaldehyde in Dulbecco's PBS (DPBS) overnight at 4°C, cryo-protected in 10% sucrose in DPBS, then embedded in Tissue-Tek medium and frozen in liquid nitrogen. Cryostat-cut four sections/animal (5 μm thick) were submitted to Hematoxylin/Eosin stain; renal damage was evaluated as tubular epithelial cell necrosis, tubular dilation, protein casts and medullary congestion (18). The alterations were semi-quantitatively graded by a pathologist blind to the nature of the experiments. The grading was performed by the following criteria: - =absent, + = barely present, ++ = moderate, +++ = severe. Expression of eNOS, e.g. the endothelial form of the constitutive NO synthase, was assessed on serial sections, with the use of a specific monoclonal antibody (BD Pharmingen, Franklin Lakes, NJ), followed by a Rabbit-anti-Mouse IgG- AlexaFluor488 (Molecular Probes, Eugene, OR). Observations were performed by using an Eclipse 55i microscope (Nikon, Tokyo, Japan), digital images acquired with DS-L1 camera and LUCIA G software (all from Nikon) and mounted using AdobePhotoshop CS software.

### Cytofluorimetry

The expression of Mac-1 was evaluated by following FACS analysis. Whole blood was incubated with 0.5 μg of FITC-conjugated CD11b monoclonal antibody (clone WT5, isotype mouse IgA, K) (Pharmingen, San Diego, CA) for 20 minutes in ice. After erythrocyte lysis, samples were run on a FACscan (Becton-Dickinson, Mountain View, CA) and gated on PMN parameters. Results are expressed as arbitrary units of mean fluorescence intensity (MFI, a.u.).

### Nitrite/Nitrate (NO2^-^/NO3^-^) determination

The rats were bled off at the aorta bifurcation level. Blood was collected in the presence of 0.065 mM citric acid (Riedel, Hannover, Germany), 0.085 mM sodium citrate (Farmitalia, Milan, Italy) and 2% glucose monohydrate (Riedel) in the blood: anticoagulant ratio of: 7:1. Samples were obtained from rats immediately after the end of each treatment or surgical procedure.

NO release was determined spectrophotometrically [19] by measuring the nitrate/nitrite (NO_2_^-^/NO_3_^-^) concentration in plasma samples from arterial non coagulated blood. Briefly, whole blood was centrifuged and plasma samples were collected, incubated for 30 min at 37°C in the presence of 0.2 U/ml *Aspergillus *nitrate reductase (Boehringer-Mannheim, Milan, Italy), 50 mM HEPES buffer (pH 7.4), 5 μM flavin adenine dinucleotide (Sigma Aldrich), and 0.1 mM NADPH (Sigma Aldrich). Then, lactate dehydrogenase (Boehringer Mannheim) and sodium pyruvate (Sigma Aldrich) were added to a final concentration of 10 U/ml and 10 mM, respectively, and the samples were incubated for 10 minutes at 37°. The Griess reagent (Sigma Aldrich) was added to the samples (100 μl), and absorbance was measured at 540 nm after 15 minutes incubation at room temperature. Standard curves with increasing concentrations of sodium nitrate and sodium nitrite were run in parallel.

### In vivo permeability assay

The assay was performed as described [20]. Briefly, the exit of albumin from vessels into the parenchyma of rat kidneys was assayed. The dye solution contained 0.4% albumin (Sigma Aldrich) and 0.5% trypan blue (Sigma Aldrich) in saline. Following laparatomy, animals were perfused with 5 ml dye-solution through the right renal artery for 10 minutes. The perfusate was drawn from the right renal vein. The right kidney was washed with saline *in vivo*, removed, weighted, suspended and homogenized in buffered phosphate solution at pH 7.4 (1 g tissue dissolved in 3 ml buffer). In treated animals, after halothane anesthesia, EDTA (40 mg/kg) was injected intravenously (through the femoral vein), followed by rat TNFα (R&D System, Abingdon, UK) (0.1 ng/g). TNFα and EDTA, alone or together, were injected 30 minutes before kidney dye perfusion.

Tissue extracts were centrifuged, the supernatants recovered and treated with 10% deoxycholic acid (sodium salt monohydrate, Sigma Aldrich) in saline, to remove lipid interference. Dye was evaluated by spectrophotometer analysis (Pye Unicam SP6-550, Cambridge, United Kingdom) at 540 nm.

### Statistics

The results are expressed as the mean ± SEM of 15 animals in each group. They were analyzed using a two way analysis of variance followed by Bonferroni t-test. The results were considered statistically significant when p < 0.05.

## Results

### Systemic hemodynamic data

In the rats studied the heart rate did not vary significantly during the experimental procedure (data not shown). To establish whether EDTA could maintain vascular homeostasis, we measured MABP in both untreated and EDTA-treated rats. EDTA treated rats displayed MABP values lower than those of untreated control and sham operated animals. To note, the increase of MABP due to Isc was significantly prevented by EDTA pre-administration (Table 1).

**Table 1:** Measure of mean arterial blood pressure (MABP) in rats

**CONTROLS SHAM**	**UNTREATMENT (mmHg)**		**EDTA pre-treatment (mmHg)**	
	
	100 ± 8		85 ± 3*	
	108 ± 11		93 ± 2*	
	
	***Before clamping***	***End Isc or Isc/R***	***Before clamping***	***End Isc or Isc/R***
	
**Isc**	104 ± 6	130 ± 5**	90 ± 2*	98 ± 7*
**Isc/R**	105 ± 6	115 ± 9	90 ± 3*	88 ± 8*

EDTA pre-administration (30 min) is able to avoid the increase of MABP induced by kidney Isc. EDTA administration reduces MABP in controls and in sham-operated rats. lsc = ischemia; lsc/R = ischemia/reperfusion.

*p < 0.05 vs. corresponding untreatment; **p < 0.05 vs Isc before clamping

### EDTA administration preserved kidneys from ischemic damage

**Figure 1:**
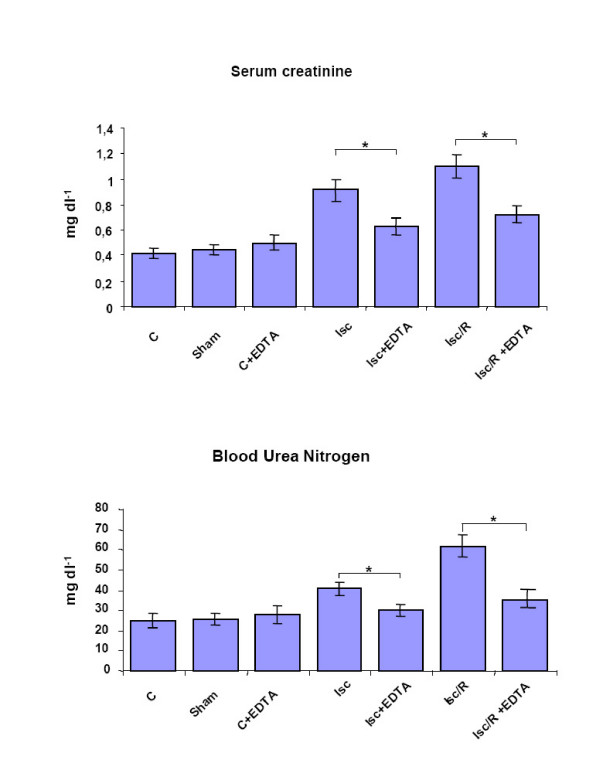
**Effect of EDTA administration on renal function after Isc and Isc/R**. Serum creatinine and blood urea nitrogen levels were measured. Rats that received intravenous injection of EDTA; 30 minutes before Isc or Isc/R induction; showed reduced levels of serum creatinine and blood urea nitrogen as compared with control rats (controls = C); lsc = ischemia; lsc/R = 60 minutes kidney ischemia followed by 60 min reperfusion. *p < 0.05.

Rats undergoing either renal Isc, obtained by clamping the right renal artery and the right renal vein for 60 minutes, or Isc followed by 60 minutes reperfusion (Isc/R), obtained by removing the clamp, were evaluated for the levels of serum creatinine and blood urea nitrogen (Fig. 1), two parameters routinely used to assess renal function. Both creatinine and urea had a significant increase after the induction of Isc and Isc/R, clearly indicating an impairment of the renal filter function. Interestingly, the administration of EDTA before Isc and Isc/R, maintained both parameters at physiological levels (Fig. 1), thus suggesting a protective role of EDTA toward the renal filter capacity.

### EDTA administration protected kidney from renal structural alterations

**Figure 2:**
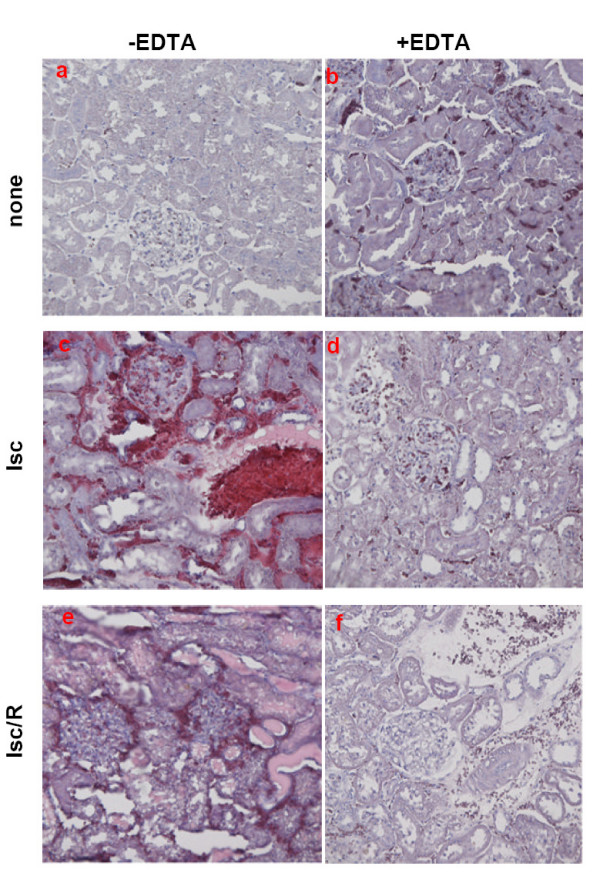
**Renal morphology**. Hematoxylin/Eosin images of differently treated rats. lsc = ischemia; lsc/R = ischemia/reperfusion. Representative cortical areas are shown. Notice the abundance of red blood cells and tubular protein casts in c and e panels in comparison with d and f (original magnification × 200).

To assess whether EDTA, administered before renal Isc or Isc/R induction, protected kidney not only from functional damage but also from structural alterations, we performed histological evaluations, aimed to determine the presence of tubular epithelial cell necrosis, tubular dilation, protein casts and medullary congestion (Fig. 2). For this, kidneys from treated rats were excised and sections were stained with Hematoxylin/Eosin, to compare their architecture with that of control kidneys (Fig. 2a). Picture relative to kidneys from EDTA-treated rats (Fig. 2b) was similar to that of control kidneys (Fig. 2a): indeed, interstitial spaces were maintained and proximal tubule as well as cortical distal segments were preserved. Kidneys from sham-operated rats did not show evidence of important modifications with respect to the controls (data not shown). Kidneys from rats undergoing Isc (Fig. 2c) showed severe renal lesions, mainly tubular, such as dilation and focal engulfment by protein casts. Glomerular and interstitial hemorrhage were also present. Some tubular cells were necrotic, whereas other appeared vacuolized. This picture worsened when kidneys were obtained from rats submitted to R (60 min) after Isc, displaying (Fig. 2e) tubular cast increase and glomerular hypertrophy. Noteworthy, kidneys from animals pre-treated with EDTA before the induction of Isc, (Fig. 2d) failed to show important renal lesions. EDTA pretreatment preserved also the architecture of kidneys submitted to Isc/R (Fig. 2f). No significant differences were evident by comparing panel d and f of Fig. 2. Pictures related to the ascending thick limb in the kidney medulla displayed interstitial hemorrhage at the end of Isc in control kidneys. On the contrary, interstitial hemorrhage was absent in kidneys from EDTA-treated ischemized rats (data not shown). The semiquantitative analysis of renal damage, which represents the mean features for each group of animals, is summarized in Table 2. The use of the eNOS inhibitor L-NAME, simultaneously injected with EDTA before the induction of Isc and Isc/R was able to block the beneficial effects induced by EDTA.

**Table 2:** Histologic evaluations of renal injury

**Rat treatment**	**Tubular necorsis**	**Tubular dilation**	**Protein casts**	**Medullary congestion**	**Glomerular damages**	**Interstitial stasis**
**Controls**	-	-	-	-	-	-
**Sham-operated**	+ *	-	-	-	-	+ *
**EDTA**	-	-	-	-	-	+
**Isc**	-	+	+ **	+/++	-	++
**EDTA+Isc**	-	-	-	-	-	+
**Isc/R**	+	++	+++	+	-	+
**EDTA+lsc/R**	+ *	++ *	+ *	-	-	+ *
**EDTA+L-NAME+Isc**	-	+	+	++	-	++
**EDTA+L-NAME+lsc/R**	+	++	+++	++	-	+

*Focal; **Big, but focal

- = absent; + = barely present; ++ = moderate; +++ = severe

Semiquantitative analysis of renal damage representative of mean features, obtained for each group of rats.

### Effect of EDTA on Mac-1 expression by PMN

To investigate a putative mechanism of action of EDTA, we considered its effect on PMN, which are largely involved in the damage associated with Isc/R [11,12].

**Figure 3:**
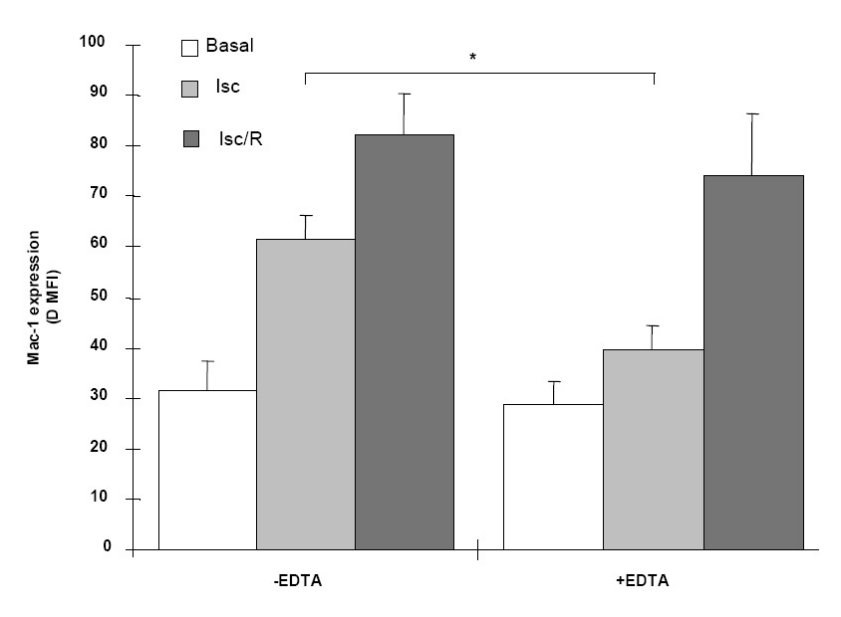
**Expression of Mac-1 by PMN recovered from rat blood**. The data represents the values; expressed as mean fluorescence intensity (MFI) (obtained by subtracting the respective value of negative control from each intensity value). lsc = ischemia; lsc/R = ischemia/reperfusion. *p < 0.05.

For this purpose, PMN, isolated from peripheral rat blood, were analyzed for the expression of the pro-adhesive molecule Mac-1 (Fig. 3); the existence of Mac-1 up-regulation is suggestive of PMN activation [21]. Mac-1 expression by PMN obtained from control rats increased significantly after Isc and Isc/R. Following EDTA pretreatment, the increase was significantly impaired in rats submitted to Isc and, at lower extent, to Isc/R.

### EDTA administration strongly influenced NO production in vivo and renal eNOS expression

**Figure 4:**
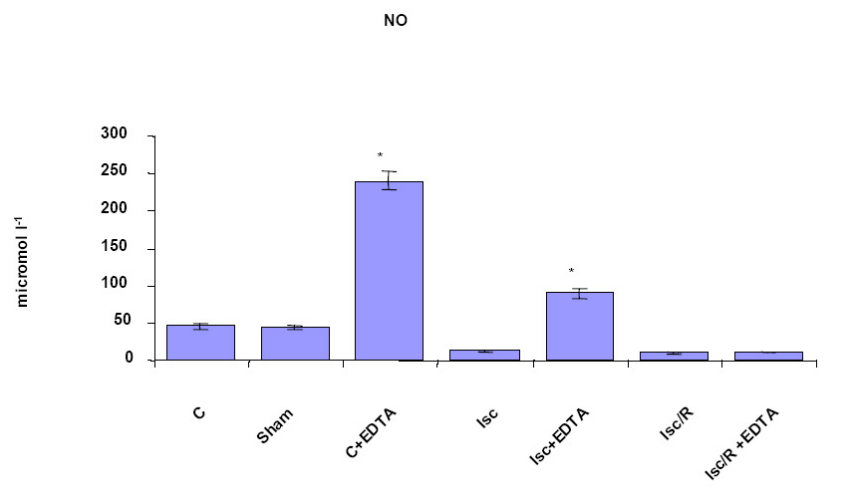
**Plasmatic NO levels**. They are expressed in μM. Rats that received intravenous injection of EDTA showed increased levels of NO as compared with controls (C). Sham = sham-operated rats. lsc = lschemia; lsc/R = ischemia/reperfusion. *p < 0.05 vs C; **p < 0.05 vs. Isc; ***p < 0.05 vs. Isc/R.

Being the expression of adhesion molecules, the adhesive and migratory pattern of leukocytes finely regulated by NO, both in physiologic and pathologic conditions [10,22-24], we then measured rat NO plasmatic levels. EDTA pre-treatment significantly increased the levels of circulating NO (Fig. 4) both in control and in ischemic rats. Conversely, post-ischemic reperfusion impaired dramatically the production of NO but was not insensitive to EDTA pre-administration: in fact NO production following Isc/R in EDTA-pre-treated rats was similar to that measured in control rats.

**Figure 5:**
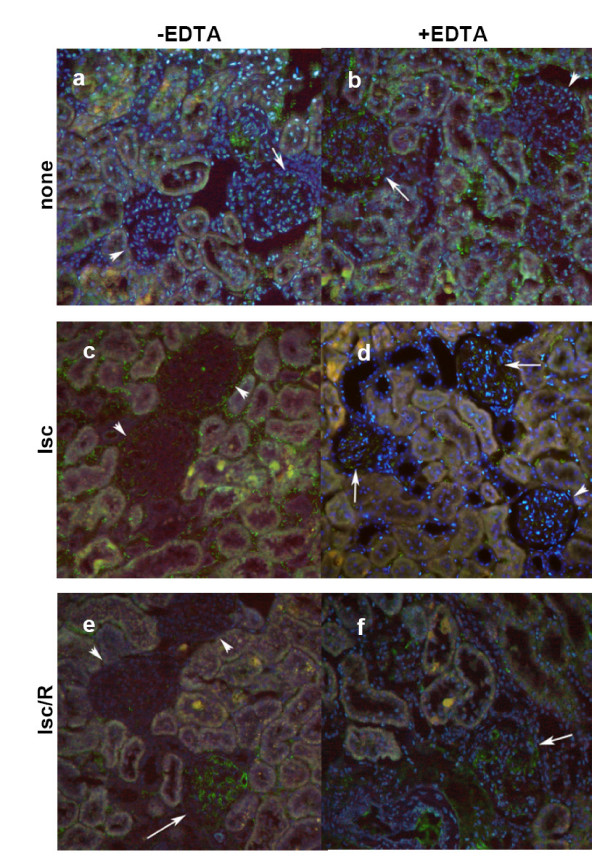
**Immunofluorescence microscopy of eNOS**. Localization of eNOS (green) on differently treated rats (lsc = ischemia; lsc/R = ischemia/reperfusion); arrows pointed to positive glomeruli; and arrowheads to negative. Nuclei were counterstained with DAPI (original magnification × 200).

As NO in vascular endothelial cells is synthesized primarily by the endothelial form of the constitutive NO-producing enzyme (eNOS), we examined the possibility that a decrease in NO bioavailability might be related to a change in the rate of expression of eNOS. The renal expression of eNOS (Fig. 5), observed in glomerular and interstitial capillaries, was slightly higher and diffuse in animals treated with EDTA (b), as compared to untreated control rats (a). Induction of short time (60 min) Isc, in control rats, produced a loss in the glomerular eNOS and an increase of its interstitial expression (c). When Isc followed EDTA treatment (d), eNOS expression was prevalently assessed inside glomerular capillaries. Kidney sections obtained following Isc/R in controls showed very low expression of eNOS both at glomerular and interstitial levels (e). Kidney sections from rats treated with EDTA before Isc/R (f) displayed fluorescence findings comparable to that of controls (a). The use of L-NAME together with EDTA before the induction of Isc and Isc/R abrogated the increase in eNOS expression due to EDTA treatment alone (data not shown).

### EDTA regulated the vascular permeability in vivo

It has been recently demonstrated that eNOS has a critical role in regulating the microcirculatory endothelial barrier function *in vivo *[25]. We investigated whether EDTA influenced the TNFα-induced vascular leakage in kidneys. Vascular leakage values (expressed as μg dye/g fresh kidney and mean ± SEM of 8 rats) are reported in Table 2. A significant increase in dye retention has been shown by kidneys of rats treated with TNFα with respect to kidneys of untreated animals (controls). EDTA treatment alone did not alter the endothelial barrier function. The concomitant administration of EDTA and TNFα resulted in the significant reduction of TNFα-induced leakage, indicating the existence of tights links among EDTA-NO-vascular protection.

## Discussion

EDTA, used in patients affected by chronic lead intoxication, improved renal function [3]. We investigated whether EDTA exerted its protective effect also toward kidneys affected by Isc or Isc/R. For this purpose, we administered intravenously EDTA 30 min before the induction of renal Isc, obtained by clamping the right renal artery and the right renal vein.

The severe renal injury induced by Isc or Isc/R was assessed both as functional impairment, through the serum creatinine and blood urea nitrogen dosages (Figure 1), and as structural alteration of tubules and glomeruli (Fig. 2). It should be noted that EDTA administration was efficient in significantly preserving renal function and in preventing structural alterations and necrotic lesions.

NO plays an important role in regulating vascular tone and improving renal blood flow [26]. We show that circulating levels of NO are increased after EDTA injection, followed or not by Isc or Isc/R (Fig. 4). NO administration could act by scavenging the ROS [6]. Indeed, the improvement of NO induced by EDTA treatment could be responsible for a reduced endothelial damage mediated by ROS. In the present study the increase of circulating NO well correlates with the expression of eNOS in kidneys from EDTA-treated rats, also when Isc or Isc/R occurred. Recent data indicates that the renal protective effects due to ischemic preconditioning are attributable to eNOS-mediated NO production [27]. In fact, it has been found that ischemic preconditioning (e.g. three cycles of 2 minutes Isc followed by 5 minutes reperfusion) was able to protect against the Isc/R-induced acute renal failure [27]. Congruously with the finding that pharmacological inhibition of NO synthesis- or disruption of the eNOS gene- significantly increases blood pressure [10,25], EDTA pretreatment has been demonstrated able to prevent the ischemic increase of MABP (Table 1).

NO modulates leukocyte adhesion in the microcirculation by decreasing the binding of PMN to the adhesion molecules E-selectin and ICAM-1 [22,24]. PMN are involved in the tissue damage due to Isc/R injury: their activation and migration in ischemic tissues is followed by release of lytic enzymes and production of ROS [11,28]. We show that Mac-1 expression, widely considered a sensitive marker of PMN activation [21], is up-regulated in rats submitted to kidney Isc and Isc/R. Treatment with EDTA prevents PMN activation in both ischemized and undergoing postischemic reperfusion rats (Figure 3). The efficacy of EDTA treatment in protecting PMN from activation is possibly mediated by the increase in NO production (Fig. 2), given that NO inhibits the increase of adhesion molecule expression [22,24]. Moreover, it has been shown that during the acute myocardial Isc/R the low level of NO increased PMN adhesion to the endothelium [23].

It is known that NO derived from eNOS is a powerful vasodilator and possesses vasoprotective effects [29]. Here we show that EDTA is able to maintain the expression of eNOS on the glomerular and interstitial capillaries after Isc and Isc/R. Several divalent cations (Mn^++^, Zn^++ ^and Fe^++^) suppressed eNOS activity in crude cell extracts and intact cells whereas Cu^++ ^increased eNOS activation [30]. So, we could argue that the removal of some divalent cations by EDTA may improve eNOS levels. In this context, the in vivo use of a divalent cation, the Cd^++^, was responsible for decreased NO concentration in rat serum [31]. Some clinical evidences support our results. Recently, chelation therapy with EDTA (also associated with vitamin B) in subjects with coronary artery disease showed a significant NO-related endothelial function improvement [32]. Analogously, iron chelation with deferoxamine infusion in cardiomyopathy patients improved NO-mediated endothelium dependent vasodilation, suggesting that iron availability contributes to impair NO action in atherosclerosis [33]. Moreover, cardiovascular protection obtained by the use of high-dose corticosteroids has been shown to be mediated by non-transcriptional activation of eNOS [34]. The role of eNOS as a trigger and mediator of isoflurane-induced delayed preconditioning in vivo has been recently reported [35].

We propose that EDTA may act through an enhancement of endothelial NO production, as previously reported for corticosteroid [34] and also for desflurane, a preconditioning agent able to protect myocardium against Isc/R injury, by favouring NO release [36].

New data suggests for EDTA the favorable antioxidant mechanism of action previously described for other chelating agents [4,7]. In fact the use of EDTA complexes with metal ions as Fe^++ ^and Cu^++ ^suppressed superoxide and hydrogen peroxide activity [37]. In addition, recently, Hininger et al. [38] showed the beneficial antioxidant effects of EDTA chelating therapy. Since oxidative stress contributes to the pathogenesis of many diseases, including cardiovascular diseases, the protection exerted by EDTA against ischemic damage could be reconducible also to its antioxidant ability.

## Conclusion

The data shows that functional and histological parameters of rat kidneys are preserved from damage due to Isc and Isc/R by EDTA treatment. These results suggest the existence of a tight loop EDTA/eNOS/NO, which on the one hand results in the loss of PMN activation and on the other hand in the maintenance of the endothelial barrier function.

## List of abbreviations used

EDTA Sodium edetate

NO Nitric oxide

eNOS Endothelial NO synthase

Isc Ischemia

Isc/R Ischemia/reperfusion

PMN Polymorphonucelar cells

L-NAME N(omega)-nitro-L-arginine methyl ester

ROS Reactive oxygen species

MABP Mean arterial blood pressure

SEM Standard error of the mean

NO_2_^- ^Nitrate

NO_3_^- ^Nitrite

Mac-1 Monocyte chemoattractant protein-1

TNFα Tumor necrosis factor

## Competing interests

The author(s) declare that they have no competing interests.

## Authors' contributions

CF performed histological and immunohisochemical analyses. AF and PT performed animal studies and collected samples. FP measured Mac-1 and performed statistical analyses. CS measured NO_2_^-^/NO_3_^-^levels. DB performed spectrophotometrical analyses and measured MABP. EF coordinated the in vitro studies. MEF coordinated the in vivo studies and wrote and edited the manuscript.

**Table 3:** Dye solution retention by rat kidneys

Treatment	**μg/g**
Controls	163 ± 8.3
+TNFα	456 ± 41.8*
+EDTA	178 ± 7.4
+TNFα+EDTA	298 ± 14.5*§

The table reports the modification of vascular permeability; following in vivo treatment with EDTA and TNFα (see Methods section). The rat right kidney was in vivo perfused with trypan blue solution; washed with saline; removed; homogenized and centrifuged. The supernatants were run on a spectrophotometer at 540 nm wavelength. The data was then expressed as μg dye retained per weight (g) of fresh kidney. Maximum dye retention (dye perfusion without washing) yielded a value of 619 ± 24.7.

*p < 0.05 vs. controls; ^§^p < 0.05 vs. TNFα

## Pre-publication history

The pre-publication history for this paper can be accessed here:


